# Woody structure facilitates invasion of woody plants by providing perches for birds

**DOI:** 10.1002/ece3.3314

**Published:** 2017-09-03

**Authors:** Chelse M. Prather, Andrew Huynh, Steven C. Pennings

**Affiliations:** ^1^ Department of Biology and Biochemistry University of Houston Houston TX USA; ^2^ Present address: Department of Biology University of Dayton Dayton OH 45469 USA; ^3^ Present address: Baylor College of Medicine Houston TX 77030 USA

**Keywords:** birds, coastal tallgrass prairie, grassland, invasive species, seed dispersal, Texas, woody encroachment

## Abstract

Woody encroachment threatens prairie ecosystems globally, and thus understanding the mechanisms that facilitate woody encroachment is of critical importance. Coastal tallgrass prairies along the Gulf Coast of the US are currently threatened by the spread of several species of woody plants. We studied a coastal tallgrass prairie in Texas, USA, to determine if existing woody structure increased the supply of seeds from woody plants via dispersal by birds. Specifically, we determined if (i) more seedlings of an invasive tree (*Tridacia sebifera*) are present surrounding a native woody plant (*Myrica cerifera*); (ii) wooden perches increase the quantity of seeds dispersed to a grassland; and (iii) perches alter the composition of the seed rain seasonally in prairie habitats with differing amounts of native and invasive woody vegetation, both underneath and away from artificial wooden perches. More *T. sebifera* seedlings were found within *M. cerifera* patches than in graminoid‐dominated areas. Although perches did not affect the total number of seeds, perches changed the composition of seed rain to be less dominated by grasses and forbs. Specifically, 20–30 times as many seeds of two invasive species of woody plants were found underneath perches independent of background vegetation, especially during months when seed rain was highest. These results suggest that existing woody structure in a grassland can promote further woody encroachment by enhancing seed dispersal by birds. This finding argues for management to reduce woody plant abundance before exotic plants set seeds and argues against the use of artificial perches as a restoration technique in grasslands threatened by woody species.

## 1 INTRODUCTION

The dominance of native and invasive woody species in historically graminoid‐dominated systems has become more prevalent across the planet (Briggs et al., 2005; Eldridge et al., 2011; Stevens, Lehmann, Murphy, & Durigan, 2017), posing a further threat to globally imperiled grassland ecosystems (Hoekstra, Boucher, Ricketts, & Roberts, 2005). Woody encroachment occurs in response to multifaceted and interacting anthropogenically influenced causes, including climate change (Sankaran et al., 2005; Eamus & Palmer, 2007; Munson et al. 2016), changes to nutrient cycles (Köchy & Wilson, 2001; Kraaij & Ward, 2006), fire suppression (Kraaij & Ward, 2006; Roques, O'Connor, & Watkinson, 2001), and overgrazing (Kraaij & Ward, 2006; Roques et al., 2001; Van Auken, 2009). Plant species responsible for woody encroachment generally decrease the diversity of grassland plant communities (Ratajczak, Nippert, & Collins, 2012) and alter abiotic conditions for native herbaceous and graminoid species (Jo, Fridley, & Frank, 2017; Siemann & Rogers, 2003).

The success of an individual woody invasive plant in a grassland depends on multiple interacting factors, including the dispersal of seeds (Boulant, Kunstler, Rambal, & Lepart, 2008), microclimatic factors such as nutrient and water availability that influence germination rates (Gabler & Siemann, 2013), and biotic factors such as the composition of surrounding plants (Siemann & Rogers, 2003) or soil organisms (Yang et al., 2013). However, as contemporary grasslands are often highly fragmented ecosystems, the establishment of many woody invasive plants may be dispersal‐limited and occur rather slowly (Briggs et al. 2005); this constraint is especially important for woody plants with large seeds that are not wind‐dispersed.

Animals, especially birds, may be an important dispersal agent of many woody plants as they can effectively transport seeds long distances from areas containing woody plants into areas lacking them (Neuenkamp, Lewis, Koorem, Zobel, & Zobel, 2016; Proctor, 1968; Sritongchuay, Gale, Stewart, Kerdkaew, & Bumrungsri, 2014). The number of stable structures for perching can limit bird use (Ferguson & Drake, 1999; Graves, Rodewald, & Hull, 2010; but see Vickery & Hunter, 1995), and as a result, increases in woody structure may enhance seed deposition as well as plant diversity by providing structure that birds use for perches (McClanahan & Wolfe, 1993; Graham & Page, 2012). The presence of woody species may especially increase the seed rain of plants with avian‐preferred fruits or seeds, especially at the times of the year when seeds have set. Thus, the presence of either exotic or native woody species may increase bird dispersal of invasive woody species (Tecco, Gurvich, Diaz, Pérez‐Harguindeguy, & Cabido, 2006). However, some studies have found that perches do not influence bird use of grasslands (Vickery & Hunter, 1995), so there is a need to better understand how wood perches influence the composition of seed rain in grasslands. This issue is of practical relevance because some authors have promoted the idea of using artificial perches to attract birds and increase seed rain into restoration projects to increase plant diversity (Guidetti, Amico, Dardanelli, & Rodriguez‐Cabal, 2016)—this approach might unwittingly promote the spread of problematic exotic, avian‐dispersed plants in grasslands. Additionally, because woody vegetation is resistant to fire, which maintains grasslands, perches might promote change of vegetation from grassland into an alternative woody state that would resist reversion to grassland (Ratajczak et al., 2017).

**Figure 1:**
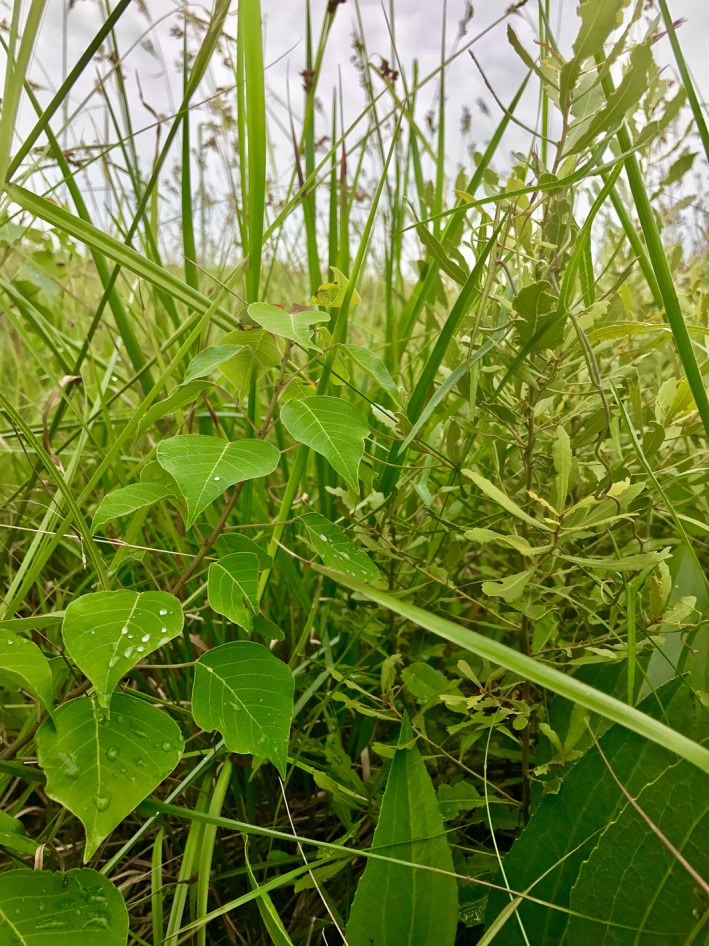
A tallow seedling (left, heart‐shaped leaves) next to a myrtle branch (right, small, elongated leaves on woody stem)

We worked in a highly endangered grassland type (Allain, Vidrine, Grafe, Allen, & Johnson, 1999), coastal tallgrass prairie, in southeastern Texas where native woody plant species exist in low abundances, but where some invasive woody plants are a severe threat. Wax myrtle, *Myrica cerifera*, is a common native woody species in these prairies. We observed that seedlings of a noxious invasive tree, Chinese tallow (*T. sebifera*), seemed more numerous in and around *M. cerifera* patches than in other types of vegetation in the summer of 2011 (Prather, personal observation). Similarly, Battaglia, Denslow, Inczauskis, and Baer (2009) found that the dispersal of *T. sebifera* seeds was higher close to *M. cerifera* thickets in freshwater marshes, but did not explore the mechanism for this result. Importantly, though, they hypothesized that, because *M. cerifera* and *T. sebifera* seem to move together across landscapes, *M. cerifera* occurrence might be a predictor of future *T. sebifera* invasions (Figure 1 shows a *T. sebifera* seedling next to a *M. cerifera* branch). We tested the overarching hypothesis that the presence of woody vegetation would facilitate the dispersal of other woody plant species by providing perches for birds, especially at certain times of the year when seeds have set and dispersal is high.

We tested three specific hypotheses: (1) *T. sebifera* seedlings are more abundant around thickets of *M. cerifera* compared to graminoid‐dominated areas; (2) the dispersal of plant seeds, especially those of woody plants that are bird dispersed, is higher under versus away from artificial wooden perches; (3) composition of seeds being dispersed differs over time between perch treatments and among habitats with different types of vegetation (graminoid dominated, shrub dominated, and *T. sebifera* dominated); dispersal should be higher in areas already dominated by woody vegetation that birds may be actively using compared to graminoid‐dominated areas.

## 2 METHODS

Our research was conducted 50 km southeast of Houston at the University of Houston Coastal Center (UHCC, 29°38′N, 95°04W). The UHCC is a ~374 ha research property previously dominated by coastal tallgrass prairie. Around 100 ha of this rare type of grassland remains, and this prairie is normally dominated by graminoids, especially *Schizachyrium scoparium*,* Andropogon geradii*,* Rhycospora cauduca,* and *Tripsacum dactyloides*. Common forb species at the site include *Euthamia letocephala*,* Helianthus angustafolius,* and *Liatris pynchostachya*, and common woody species include the natives *M. cerifera* and *Baccharis halimifolia,* and the invasive exotic Chinese tallow, *T. sebifera*. Forests dominated by *T. sebifera* and other woody invasive species with sparse native trees, such as *Celtis laevigata*, are now the most abundant habitat on the property and are a common habitat type throughout the Gulf Coast (Bruce, Cameron, Harcombe, & Jubinsky, 1997). These woody species threaten prairie remnants where managers use regular mowing or burning to limit their spread.

To test hypothesis one, that *T. sebifera* individuals were more common throughout or around the outside perimeter of thickets of *M. cerifera* than in graminoid‐dominated areas, we compared the density (the number per m^2^) of *T. sebifera* individuals in paired, similarly sized *M. cerifera* patches and graminoid‐dominated patches in May of 2012 (*n *=* *125 pairs of patches). We chose patches by randomly first locating one *M. cerifera* patch and a randomly selected graminoid‐dominated area at least 1 m outside of the *M. cerifera* patch. Basal area of each *M. cerifera* patch was calculated by measuring the radius from the center of the *M. cerifera* patch to 0.3 m outside of the patch; *T. sebifera* seedlings in graminoid‐dominated areas were counted in a patch of the same basal area. We then walked a randomly chosen distance and located the next *M. cerifera* patch (typically at least 20 m away) and repeated the process.

To test hypothesis two, that seed dispersal is higher under woody perches, we measured how woody perches influenced the amount of seed rain (number of seeds per month) and the number of seeds in different plant functional groups every month by deploying artificial wooden perches (r‐shaped, 0.5‐cm‐diameter dowels, 1 m tall with a 10‐cm horizontal perch) constructed from untreated pine. These perches are as tall as existing woody vegetation, but likely a little more robust. Paired seed traps were placed on the ground underneath an artificial perch or ~2 m away without an artificial perch. We made seed traps by securing mesh hardware cloth (1 cm) over rectangular plastic containers (29.85 cm × 19.69 cm × 12.7 cm high). Mesh was folded underneath the top edges of the seed traps; seeds fell through the mesh hardware cloth into the container, and the hardware cloth kept other animals (e.g., rodents) from getting into the traps and eating the seeds. Each pair of seed traps was located at least 4 m from the next pair, and traps were put on the soil surface.

To test hypothesis three, that the effects of perches on seed rain over time composition are a function of background vegetation, we deployed these seed traps into three different types of vegetation: graminoid‐dominated areas, *M. cerifera* patches, and areas of abundant *T. sebifera* saplings. Five pairs of seed traps were placed in each of three replicate locations of each habitat, for a total of 180 seed traps (n = 5 replicates* perch presence or absence * three replicate locations * three vegetation types = 90 pairs of seed traps). Seed traps were deployed from June 2012 to March of 2013 to span the period during which most plants at the site were producing seeds. We removed all seeds from seed traps every month, sorted them to morphospecies, and counted the number of each morphospecies in each trap per month. We identified 23 of 33 morphospecies we observed to species by consulting keys of local vegetation (grasses—Hatch, Schuster, & Drawe, 1999; woody vegetation—Vines, 1977; Wrede, 2010), and plants were put into functional groups.

We documented rodent activity by looking for rodent feces in seed traps or bite marks on seeds. Rodent activity was rare (observed on only 29 occasions of over 540 individual collections from seed traps), and perches did not alter rodent activity. We did, however, regularly observe birds using the perches and found bird droppings only in seed traps that were located underneath perches.

To test for differences in the abundance of *T. sebifera* seedlings inside versus outside *M. cerifera* patches, we used a paired *t* test. To test for differences in total number of seeds and overall species richness, we conducted repeated measures GLMs for the total number of seeds and seed species richness. To test for differences in the number of seeds of each functional group (graminoids, herbaceous plants, woody plants, and unknown groups), we used repeated measures, multivariate GLMs (with date as the repeated measure, and perch presence or absence and vegetation type as fixed factors). All possible interactions were examined because mechanistically each interaction could be important. For example, the interaction date * perch * vegetation could arise if seed rain changes under perches differently in different vegetation types. Post hoc means comparisons for differences between vegetation types were performed using Tukey's test. To determine if particular species were driving responses of functional groups to perches, for any functional group with significant effects relevant to our hypotheses (any significant main effects of or significant interactions with perch presence), we conducted follow‐up repeated measures, multivariate GLMs as described above with numbers of seeds from individual species that composed each functional group. To look at how seed composition of different plant functional groups changed over time with and without perches, we used PCA and extracted values for any principle components with eigenvalues over 1. We examined how seed composition changed graphically by plotting the average PCA values (±*SE*) for each date for seed composition underneath perches or not. Data were analyzed in SAS.

## 3 RESULTS

**Figure 2:**
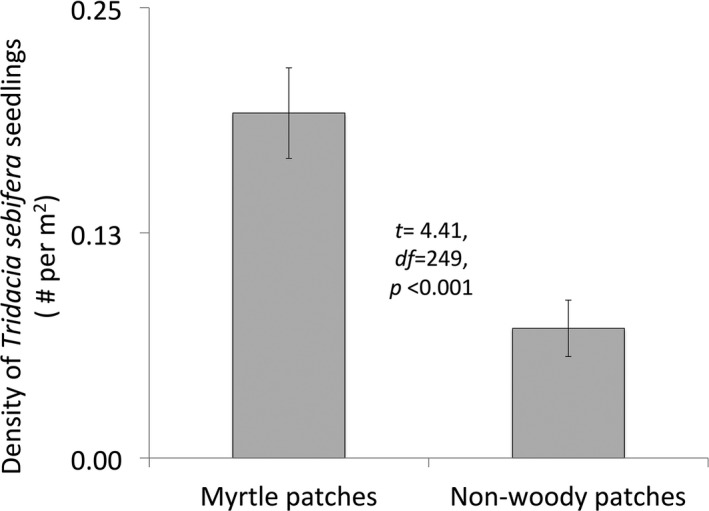
The number of *Tridacia sebifera* seedlings found in myrtle patches versus nonwoody patches

**Figure 3:**
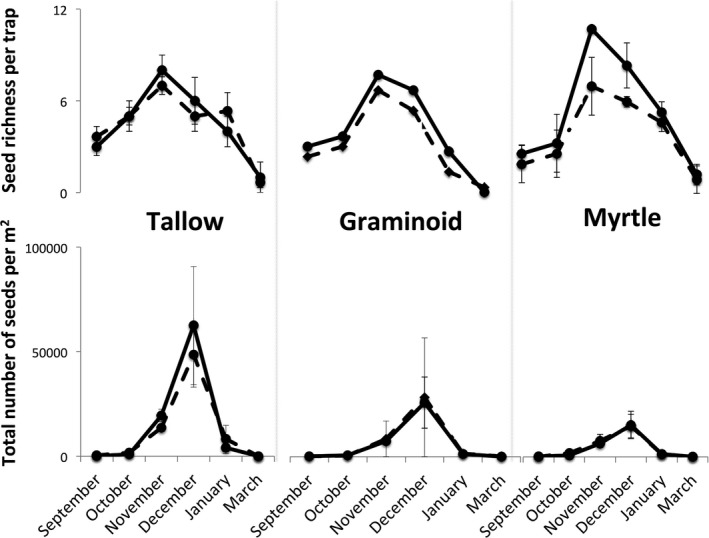
Seed species richness (top panels) and abundance (bottom panels) per seed trap in three vegetation types. Solid lines were seed traps underneath perches, and dashed lines were seed traps not underneath perches. Perches increased the richness of seeds in myrtle‐dominated patches in months when seed rain was highest (Table 1)

*Tridacia sebifera* seedlings were over twice as dense underneath *M. cerifera* thickets as in graminoid‐dominated areas of the same size (Figure 2; *t = *4.41, *df *=* *249, *p *<* *.001). Both the abundance and species richness of seeds found in seed traps changed over time in all three types of vegetation: overall seed abundance was highest in December in all three vegetation types (date: *F *=* *6.85, *p *=* *.006) and the species richness of seeds was highest in November in all three vegetation types (Table 1, Figure 3). The effects of date, however, were not as strong in every type of vegetation: tallow‐dominated sites had more total seeds and a stronger effect of date, and myrtle‐dominated sites had fewer seeds and a dampened effect of date (date × vegetation type: *F *=* *16.63, *p *<* *.001). Myrtle‐dominated sites, however, had more different species of seeds than other sites, especially in November (date × vegetation type: *F *=* *5.31, *p *=* *.007). When broken into plant functional groups, graminoids and herbaceous plants followed the patterns described above, but woody plants did not, with the number of seeds not differing across date or vegetation type.

**Table 1:** Summary results for analyses of seed richness and total seed abundance (a separate univariate GLM for each), the number of seeds from each plant functional group, and for each woody species of vegetation found (a separate MANOVA for each). For all tests, date was a repeated measure, while perch and vegetation type were fixed factors. “(I)” indicates invasive species. Bold indicates significant *p*‐values

	Date		Veg		Perch		Date × Veg		Date × Perch		Veg × Perch		Date × Veg × Perch	
	*F*	*p*	*F*	*p*	*F*	*p*	*F*	*p*	*F*	*p*	*F*	*p*	*F*	*p*
Seed richness	17.36	**<.001**	1.21	.36	2.55	.20	5.31	**.007**	3.01	**.007**	4.46	**.04**	0.32	.97
Total seed abundance	6.85	**.006**	2.13	.17	0.50	.661	16.64	**<.001**	0.65	.67	0.99	.41	0.21	1.00
Graminoids	19.64	**<.001**	8.62	**<.001**	0.14	.71	3.78	**.001**	0.18	.97	0.22	.81	0.21	1.00
Herbs	17.52	**<.001**	7.22	**.001**	1.57	.21	1.18	.32	0.35	.88	0.13	.88	0.16	1.00
Unknown dicots	3.27	**.01**	0.46	.64	2.82	.10	0.96	.49	1.49	.20	0.44	.65	0.88	.56
Woody plants	2.17	.07	2.19	.12	10.7	**.002**	0.77	.66	1.81	.12	1.81	.17	0.55	.85
*Berchemia scandens (I)*	3.64	**.005**	2.09	.13	10.25	**.002**	0.75	.68	3.64	**.01**	2.09	.13	0.75	.68
*Celtis laevigata*	1.20	.32	0.96	.39	2.88	.09	0.72	.70	1.20	.32	0.96	.39	0.72	.70
*Myrica cerifera*	1.25	.30	0.88	.42	0.75	.39	0.88	.56	0.75	.59	1.12	.33	1.12	.36
*Tridacia sebifera (I)*	2.75	**.03**	0.79	.46	8.73	**.004**	0.77	.66	2.44	**.04**	0.18	.83	0.60	.81

**Figure 4:**
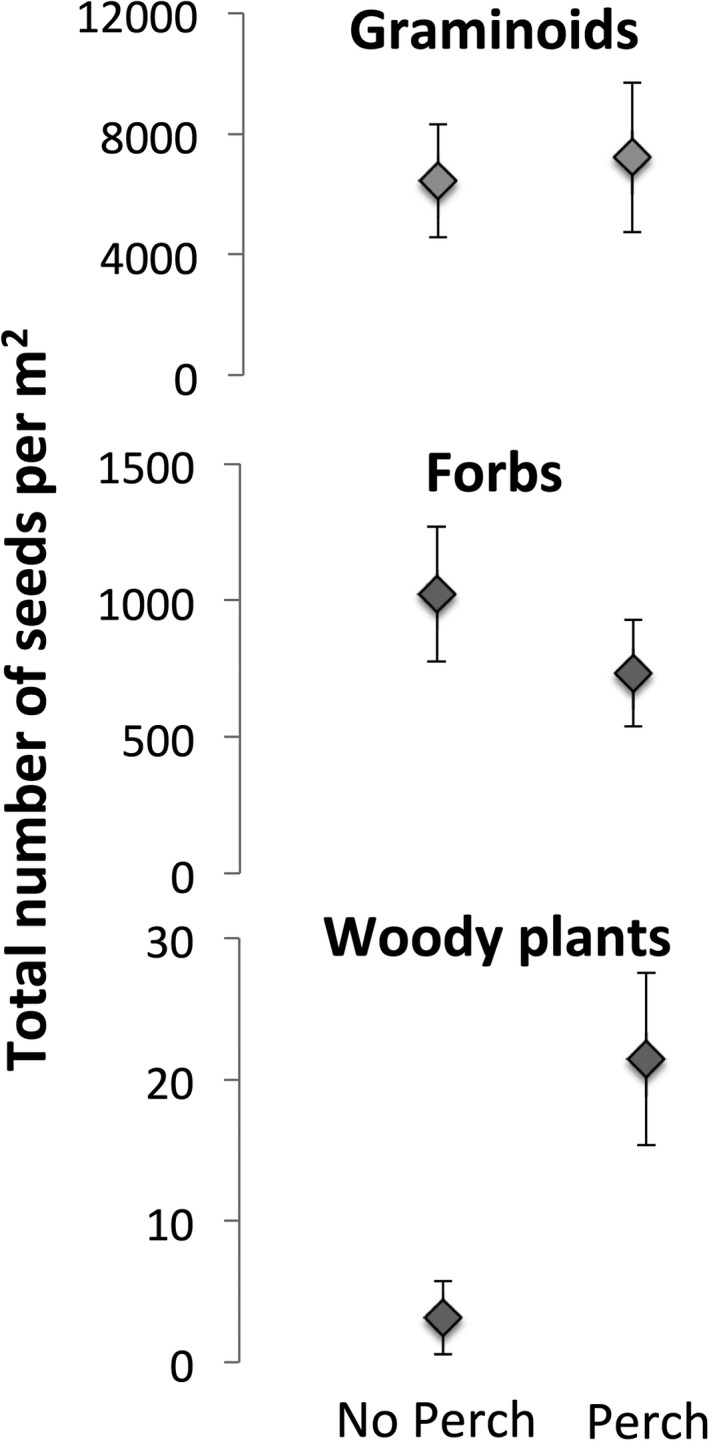
The total number of seeds of different functional groups found in seed traps underneath perches and not underneath a perch. Woody plants were the only functional group that responded to perches

**Figure 5:**
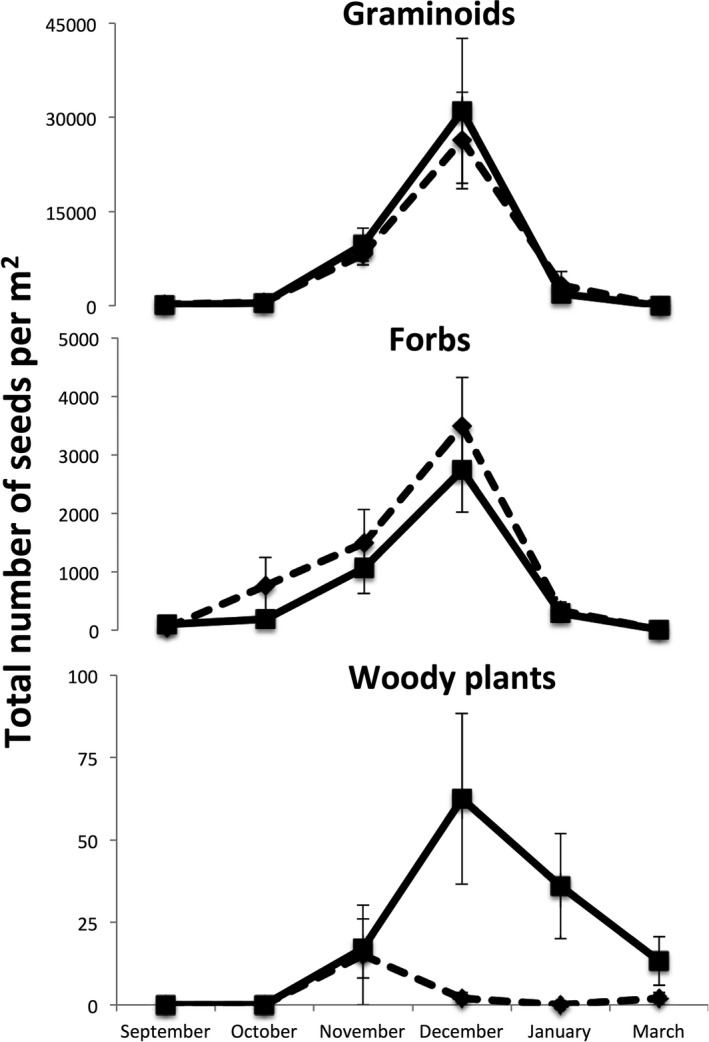
The total number of seeds from each plant functional group observed underneath perches (solid line) and not underneath perches (dashed line). Perches altered the number of woody plant seeds in months when seed rain was highest but did not alter graminoid or forb seed rain (Table 1)

**Figure 6:**
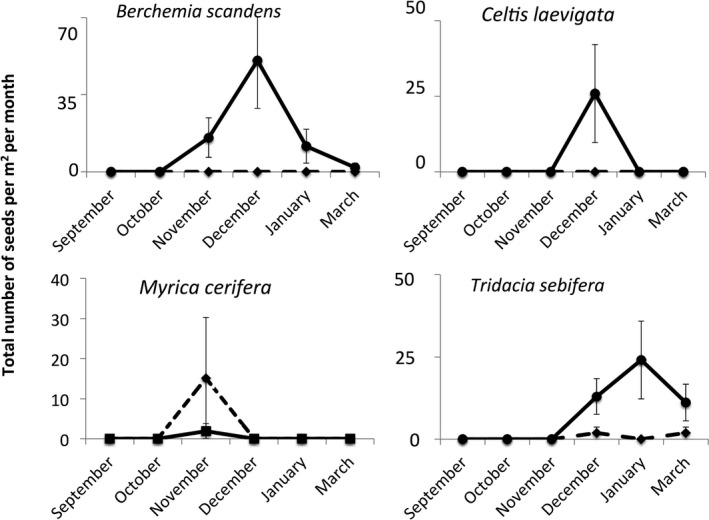
The number of seeds of four woody species of plants that we observed underneath perches (solid line) and not underneath perches (dashed lines). Perches altered the number of seeds from two invasive woody plant species (*Berchemia scandens* and *Tridacia sebifera*) in months when seed rain was highest (Table 1)

Perches increased the number of woody plant seeds sevenfold in all vegetation types (Figure 4, Table 1, perch: *F *=* *10.65, *p *=* *.002) and enhanced species richness in wax myrtle vegetation (Figure 3; vegetation type × perch: *F *=* *4.64, *p *=* *.007), but did not affect the total number of seeds collected (perch: *F *=* *0.49, *p *=* *.66; all interactions *p* > .05), or the species richness of seeds found (perch: *F *=* *2.55, *p *=* *.20). Moreover, the numbers of graminoid seeds and herbaceous seeds were not affected by perches in any type of vegetation (Figure 5, Table 1). The increase in woody plant seeds was driven by two invasive species, with perches increasing the number of *T. sebifera* around 20 times and *Berchemia scandens*, around 30 times while perches did not affect the number of seeds from native woody plant species (Table 1, Figure 6).

**Figure 7:**
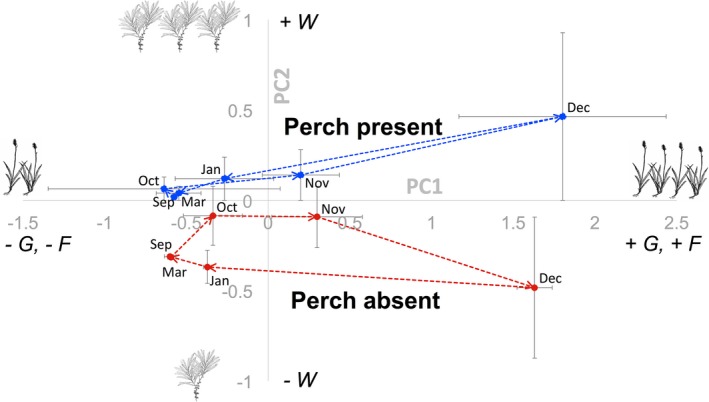
Composition of seed rain changes over time as depicted by plotting PC1 and PC2. When perches are present, the composition of seed rain becomes much more dominated by woody plant species across all vegetation types over time compared to when perches are not present

Perches greatly altered the species composition of seeds, and these differences changed over time (Figure 7). The functional groups of seeds fell onto two principle components: PCA 1 (35% of variance; 1.4 eigenvalue) that indicates grasses (0.82) and forbs (0.83) and PCA 2 (27% of variance, 1.1 eigenvalue) that indicates woody vegetation (0.71) and unknown dicots (0.74). Seed rain under perches was always more dominated by woody plants than seed rain in areas without perches (Figure 7). The magnitude of the perch effect varied seasonally, with the greatest differences between treatments in December when seed rain was at its peak.

## 4 DISCUSSION

**Figure 8:**
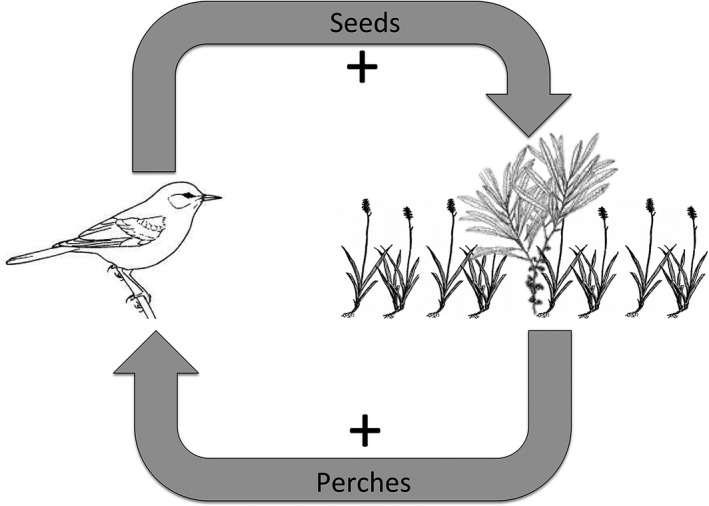
Conceptual diagram of a positive feedback loop created by bird dispersal of seeds into and around a woody thicket

Woody encroachment is a severe threat to prairies globally, and thus an understanding of the mechanisms that facilitate this process is crucially important for informing management of the remaining prairie remnants. Our data suggest that a positive feedback loop exists between existing woody structure and animal dispersal of woody species that may enhance woody encroachment into this coastal tallgrass prairie (Figure 8). In particular, we verified that more *T. sebifera* seedlings were present around myrtle patches (Hypothesis 1). Although perches did not increase the total number of seeds delivered by birds, they did enhance the delivery of woody seeds (Hypothesis 2). Additionally, perches altered seed composition by increasing the seed rain of two invasive of woody plant species in months when seed rain was highest (Hypothesis 3). Most likely, woody structure provides perches where animals, such as birds, either drop seeds while feeding (i.e., messy eating) or pass them in their feces. This positive feedback loop may be further enhanced by increased growth and survival of woody seedlings underneath woody plants, as has been shown for *T. sebifera* (Siemann & Rogers, 2003), but this “nurse plant” hypothesis was not explicitly tested in this study. It has recently been suggested that perches may be used in a wide array of ecosystems as a method to increase seed pressure of native species to enhance restoration and recovery of ecosystems (Guidetti et al., 2016). We argue, however, that in grasslands threatened by woody plants this is a risky process that could increase the seed pressure of woody invasives, particularly those dispersed by animals.

Two woody species of plants were solely or nearly always only dispersed under perches: *Tridacia sebifera* (an invasive tree) and *B. scandens* (a native but invasive woody vine in grasslands). *Tridacia sebifera* has a substantial outer seed coating that is high in lipids and proteins (Potts & Bolley, 1946; Huoran & Pengxin, 1991) that makes it attractive to birds, especially during the food‐scarce winter. Accordingly, *T. sebifera* is dispersed by a diverse array of generalist birds (Renne, Barrow, Johnson Randall, & Bridges, 2002; Samuels, 2004). Birds, including turkey and bobwhite, also regularly eat *B. scandens* fruits (Miller & Miller, 1999). Because birds often feed and defecate while on perches, the presence of woody vegetation—or in our case artificial perches—is likely to increase the seed rain of woody species, and if seeds are able to persist and germinate, accelerate woody encroachment into these coastal prairies.

Although, as an alternative hypothesis, rodents (hispid cotton rats, *Sigmodon hispidus*; fulvous harvest mice, *Reithrodontomys fulvescens*; and rice rats, *Oryzomys palustris*; Kincaid & Cameron, 1982) could also be dispersers of these three plant species, rodent activity (e.g., teeth marks on seeds, feces.) was rare and did not depend on perches or habitat type. In contrast, we did notice significant bird activity on perches—we observed birds using the perches directly on several occasions and found bird droppings only in seed traps underneath perches. It would be of interest in future work to better document which bird species were dispersing seeds, and whether rodents were doing any dispersal, because these details of the mechanism(s) might provide additional insight into the positive feedback loop that we observed between woody structure and seed pressure.

Grasslands are often highly managed systems. The positive feedback loop that we argue can exist between woody plant abundance and enhanced dispersal of woody plants indicates that grassland managers should be vigilant to avoid situations that may result in temporary increases in woody species (e.g., depending on the particular grassland: droughts, fire suppression, overgrazing, low‐mowing frequency), because any increase in woody species is likely to promote the further establishment of undesirable woody plants. If changing climatic conditions, like increased variability in precipitation, promote an increase in woody structure, this positive feedback loop between woody structure and increase in seed pressure due to animal dispersal may become even more important. Quick management of increases in woody plants may be especially important for grasslands that are under threat by a woody invasive species whose seeds can persist in the seed bank for long periods of time, like those of *T. sebifera* (Cameron, Glumac, & Eshelman, 2000).

Our results also have implications for the best seasonal timing of management activities. In particular, control of woody vegetation in Texas may be most effective in limiting dispersal of *T. sebifera* seeds if the woody vegetation is removed before December, when *T. sebifera* seeds begin to be dispersed (Figure 3). If woody structure is not lessened or removed before birds disperse potentially noxious seeds into a prairie, management efforts may be much less effective due to larger annual increments of exotic seeds into the seed bank.

Finally, it has been suggested that artificial perches may enhance grassland restoration efforts by increasing seed pressure (Guidetti et al., 2016). Here, we did not see that the total number of graminoid, forb, or native woody plant seeds was enhanced by perches. However, we did find that artificial perches increased the species richness of seeds found in seed traps in certain types of vegetation; thus, using artificial perches may be an appropriate and beneficial restoration tactic in areas where woody invaders are not a threat. Our results, however, suggest that in grasslands threatened by woody encroachment or woody exotics providing artificial perches may undermine restoration efforts by increasing the supply of seeds of woody species. Thus, artificial perches should only be used in appropriate circumstances, and the outcome of their use monitored to insure that they are beneficial rather than deleterious to grassland condition.

## CONFLICT OF INTEREST

None declared.

## AUTHOR CONTRIBUTIONS

CMP, AH, and SCP conceived the ideas and designed the methodology; AH collected the bulk of the data with help from CMP; CMP analyzed the data and led the writing of the manuscript. All the authors contributed critically to the drafts and gave final approval for publication.
